# Stroke Risk in Head and Neck Cancer: A Meta‐analysis of Reconstructed Individual Patient Survival Data

**DOI:** 10.1002/ohn.1249

**Published:** 2025-04-07

**Authors:** Eda Liew, Jing Xuan Tan, Chen Ee Low, Doreen Shu Lin Goh, Esther Yanxin Gao, Yao Hao Teo, Emilie C.M. de Groot, Jasper Senff, Ching‐Hui Sia, Leonard Leong Litt Yeo, Anna See, Benjamin Kye Jyn Tan, Benjamin Yong‐Qiang Tan

**Affiliations:** ^1^ Yong Loo Lin School of Medicine National University of Singapore Singapore Singapore; ^2^ Department of Otorhinolaryngology–Head and Neck Surgery Singapore General Hospital Singapore Singapore; ^3^ Department of Otolaryngology–Head and Neck Surgery Massachusetts Eye and Ear Boston Massachusetts USA; ^4^ Department of Neurology Massachusetts General Hospital Boston Massachusetts USA; ^5^ Division of Neurology Department of Medicine, National University Hospital Singapore Singapore

**Keywords:** head and neck neoplasms, radiation therapy, squamous cell carcinoma of head and neck, stroke

## Abstract

**Objective:**

Although previous studies suggest an increased stroke risk in head and neck cancer (HNC) survivors, the risk with various treatment modalities, including radiotherapy, is less certain. This study investigates stroke incidence and risk in HNC patients, including how different treatments influence stroke risk.

**Data Sources:**

A literature search of PubMed, Scopus, and Embase was conducted.

**Review Methods:**

We included all primary studies assessing stroke as an outcome in HNC patients aged 18 and older, regardless of cancer subtype or treatment modality. Incidence rates were pooled by reconstructing individual patient time‐to‐event data from survival curves. Random‐effects meta‐analyses were employed to compare stroke risk between HNC patients, healthy controls, and treatment groups.

**Results:**

In total, 15 studies (N = 2,295,447 patients) were included in the analyses. Among surviving HNC patients, stroke occurred at a rate of 1% per year (10% at 10 years and 15% at 15 years cumulatively). Meta‐analyses showed that HNC patients had a significantly higher stroke risk compared to healthy controls (hazard ratio [HR] = 1.45; 95% CI: 1.27‐1.65; *I* ^2^: 20%). Among HNC patients, radiotherapy alone increased stroke risk compared to surgery alone (HR = 1.66; 95% CI: 1.35‐2.03; *I* ^2^: 0%). Patients who received any form of radiotherapy had higher stroke risk compared to those without (HR = 1.47; 95% CI: 1.29‐1.68; *I* ^2^: 60%). Patients with definitive chemoradiotherapy had heightened stroke risk compared to patients who received definitive surgery (HR = 1.28; 95% CI: 1.09‐1.49; *I* ^2^: 86%).

**Conclusion:**

Patients with HNC face an elevated stroke incidence and risk, especially those treated with radiotherapy. This underscores the need for surveillance and tailored preventive strategies to reduce stroke risk in this vulnerable population.

Head and neck cancers (HNCs) and stroke are major causes of morbidity and mortality. Globally, HNCs are the seventh most common cancer, with an annual incidence of 890,000,^1^ whereas stroke is the second leading cause of death, with an annual mortality rate of 6.6 million.^2^ HNC incidence is also on the rise globally, with an estimated annual percentage change of 0.35.^3^ This upward trend is likely to continue, especially with the increasing incidence of head and neck squamous cell carcinoma (HNSCC) from smoking, alcohol, and betel nut chewing in developing countries,^4^ as well as human papillomavirus (HPV)‐related HNSCC commonly seen in the oropharynx in developed countries.^5^

Recent evidence suggests a link between HNC and stroke. Cancer survivors are well‐known to have an increased incidence of stroke related to hypercoagulability, tumor occlusion of vessels, or complications of treatment.^6^ In HNC survivors, this is especially pertinent, as treatment necessitates surgical manipulation and radiotherapy to the head and neck vessels, including the common carotid, internal carotid, and other major vessels from which the brain derives its blood flow. Radiotherapy accelerates atherosclerosis,^7^ surgical manipulation may dislodge emboli, and chemotherapy further induces systemic endothelial damage.

Although previous meta‐analyses have examined the stroke risk in HNC survivors after radiotherapy,^8^, ^9^ these meta‐analyses had important limitations in design which weakened their reliability. For instance, they did not assess the risk of bias in each study or the overall quality of evidence^8^ and did not assess or adjust for publication bias.^9^ These are crucial steps in systematic reviews that should be performed. Moreover, the papers only reported the risk of stroke in patients who underwent radiotherapy and did not compare the risk of stroke in HNC patients subject to other treatment modalities. With the recent publication of multiple large cohort studies, including nationwide cohort studies,^10^, ^11^, ^12^, ^13^, ^14^ it is timely to re‐examine the available evidence.

To address this, we conducted a thorough systematic review and meta‐analysis to assess the incidence and risk of stroke in patients with HNC, specifically examining how different treatment modalities influence stroke risk.

## Methods

### Protocol and Guidance

This systematic review is reported according to Preferred Reporting Items for Systematic Reviews and Meta‐analyses (PRISMA) reporting guidelines, presented in Supplemental Table S1, available online. The study protocol was registered prospectively on the International Prospective Register of Systematic Reviews, PROSPERO (Reference: CRD42024563925).

### Data Sources and Search Strategy

A literature search was performed in PubMed, Embase, and Scopus. The search strategy combined search terms for stroke, cerebrovascular disease, and head and neck cancer. The search strategy was translated between each database. Examples of the full strategies are available in Supplemental Table S2, available online.

### Study Selection: Inclusion and Exclusion Criteria

Two reviewers independently screened the titles and abstracts, followed by full texts, of all studies for eligibility according to the strict inclusion and exclusion criteria. Discrepancies were resolved by adjudication of the two reviewers with a third independent reviewer.

**Figure 1:**
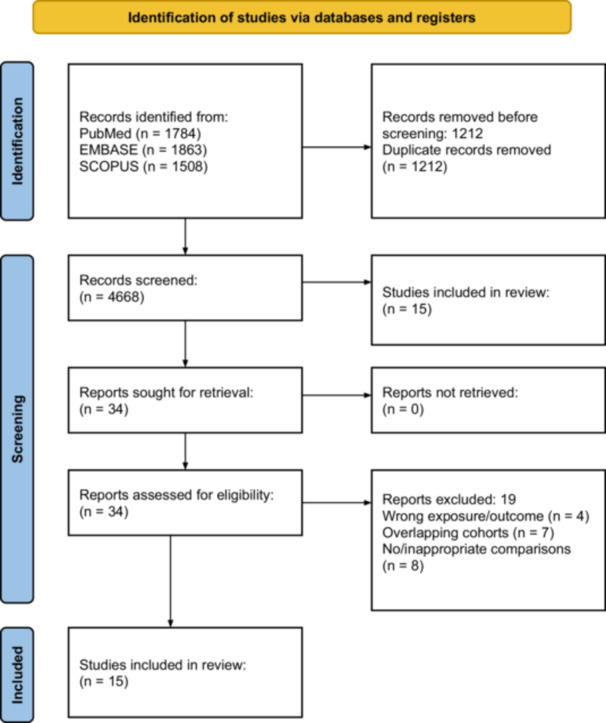
Preferred Reporting Items for Systematic Reviews and Meta‐analyses flowchart.

Our review included English‐language studies published since inception to April 25, 2024, that investigated stroke incidence among adult patients (aged at least 18 years) with primary HNC. We included descriptive studies that reported the incidence of stroke in HNC patients via survival or cumulative incidence curves. We also included analytical time‐to‐event studies that reported hazard ratios (HRs) comparing stroke incidence in HNC patients versus healthy controls, or between different HNC treatment modalities. We excluded studies if they: investigated stroke mortality; studied patients where the primary cancer did not originate from the head and neck; used unadjusted analytical estimates or count outcomes (eg, risk ratio, odds ratio). The selection process is shown in Figure 1.

### Data Extraction and Organization

The relevant data were extracted by two reviewers with all extraction cross‐checked by a third reviewer. The data extracted included the aim of the study, demographics (mean age, country of study, and sample size), and characteristics of patients with HNC, such as subtype of HNC (nasopharyngeal, laryngeal, oropharyngeal, oral cavity, hypopharynx, and salivary gland), baseline characteristics (hypertension, diabetes, hyperlipidemia, ischemic heart disease, stroke, smoking, atrial fibrillation, and chronic kidney disease), and treatment types (chemotherapy, surgery, and radiotherapy). We extracted the main findings of the study, including the incidence of stroke and HRs.

### Statistical Analysis

In the primary analysis, we first reconstructed time‐to‐event individual patient data (IPD) from digitized survivor or failure curves by solving the inverted Kaplan‐Meier (KM) product limit equations using iterative numerical algorithms.^15^ To aid the reconstruction of IPD, we extracted step function values and timings as *x*‐ and *y*‐coordinates from available digitized vector and raster images of survivor or failure curves using a semiautomated web‐based tool (WebPlotDigitizer, version 4.5). We then evaluated the accuracy of reconstruction using published methods.^16^ We performed a nonparametric, two‐stage, inverse variance meta‐analysis with random effects to retrieve a distribution‐free summary survival curve.^17^, ^18^ This method expands the product limit estimator of survival for aggregated survival data. The reconstructed KM curves can be found alongside the original KM curves in Supplemental Figure S6, available online, alongside the demonstration that the estimated survival probabilities correspond with those of the read‐in probabilities. As the competing event of death was not accounted for, the incidence of stroke reported at 1‐year intervals relates to the probability of recovery for patients who remain alive before the time. The extension of DerSimonian‐Laird methodology for multiple outcomes was applied to account for between‐study heterogeneity.^17^

In the secondary analysis, we pooled HRs (comparing the risk of stroke among HNC patients vs controls, or between different treatment modalities for HNC) using inverse variance, random‐effects meta‐analyses. *I* ^2^ and *τ* ^2^ statistics were used to analyze between‐study heterogeneity, with an *I* ^2^ value of <30%, 30% to 60%, and >60% indicating a low, moderate, and high proportion of variability due to between‐study heterogeneity.^19^ Sensitivity analysis was performed using leave‐one‐out influence analysis.^20^ Funnel plot asymmetry, which may suggest publication bias, was evaluated qualitatively via visual inspection and quantitatively via Egger's regression test^21^ and the trim‐and‐fill method (R0 estimator, fixed random‐effects models) to reestimate the pooled effect size after imputing potentially missing studies.^22^, ^23^

All analyses were conducted on R (version 4.1.0) using the *IPDfromKM*, *MetaSurv*, *meta*, and *metafor* packages. A two‐sided *P* value of <.05 was considered statistically significant, unless otherwise specified.

### Risk of Bias

The risk of bias was assessed using the Newcastle Ottawa Scale for cohort studies.^24^ Studies were graded as having a low, moderate, or high risk of bias. This appraisal was performed by two reviewers independently.

## Results

### Risk of Bias, Publication Bias, and Sensitivity Analyses

The risk of bias of the 15 studies included in the systematic review and meta‐analysis, as scored with the Newcastle Ottawa Scale, is presented in Supplemental Table S3, available online. Out of the 14 included studies, nine studies were rated as “good,”^13^, ^14^, ^25^, ^26^, ^27^, ^28^, ^29^, ^30^, ^31^ four studies^11^, ^32^, ^33^, ^34^ were rated as “fair,” whereas one study was rated as “poor.”^35^

Sensitivity analyses using funnel plots, trim‐and‐fill, and Egger's test (intercept = 0.29; 95% CI, −1.46 to 0.58; *t* = −0.85; *P* = .5077) revealed possible asymmetry (Supplemental Figures S1‐S4, available online). Trim‐and‐fill imputed three missing studies (Supplemental Figure S2, available online) with minimal change to the pooled effect size (HR, 1.38; 95% CI, 1.24‐1.53; *I* ^2^ = 81%; n = 9 + 3). Leave‐one‐out analyses showed that there was no single study that altered the overall results (Supplemental Figure S5, available online).

### Results

The PRISMA flowchart is presented in Figure 1. The search identified 5155 studies, of which articles were screened by title and abstract, following duplicate removal and exclusion of irrelevant studies. A total of 15 studies published between 2002 and 2024, with a total of 2,295,447 patients, were included in the review.^11^, ^13^, ^14^, ^25^, ^26^, ^27^, ^28^, ^29^, ^30^, ^31^, ^32^, ^33^, ^34^, ^35^, ^36^ All were observational population‐based retrospective cohort studies, based in different countries. One study was based in Singapore,^13^ two in Canada,^35^, ^36^ two in the Netherlands,^32^, ^34^ two in South Korea,^14^, ^33^ three in Taiwan,^11^, ^29^, ^30^ and five in United States.^25^, ^26^, ^27^, ^28^, ^31^

One study was specific to nasopharyngeal cancer,^30^ whereas the other 14 studies reported on various subtypes of HNCs. The mean age range for all studies was from 50.1 to 76 years. Three studies used the general population as a point of comparison.^13^, ^30^, ^31^ Two studies^14^, ^29^ compared against a reference cohort matched for age and gender, whereas one study^30^ used appendectomy patients as a surrogate measure of the general population. One study^11^ used an unmatched general population cohort of randomly selected individuals aged 20 to 85 years. The remaining eight studies compared within the cohort of patients diagnosed with HNC, with two studies^28^, ^36^ comparing against HNC patients who underwent surgery, two studies^26^, ^35^ comparing against HNC patients who did not use statins, one study^27^ comparing against HNC patients who underwent nonsurgical therapies including chemoradiotherapy or radiotherapy, whereas the other three studies^28^, ^33^, ^34^ compared against HNC patients who did not undergo radiotherapy. The main characteristics of these 15 studies included are summarized in Supplemental Table S4, available online. The duration of follow‐up of these studies ranged from 2 months to 20 years. The sites and stages of HNC of the population in the included studies, where available, are found in Table 1.

**Table 1:** Sites and Stages of HNC in the Included Studies

		HNC type, %							HNC stage, %			
Author	Publication year	NPC	Laryngeal	Oropharyngeal	Oral cavity	Hypopharynx	Salivary gland	Others	1	2	3	4
Addison	2018	7.8	10.9	46.5	0	6.2	0	32.8	78.8			21.2
Arthurs	2016	6.7	31.6	25.1	27.1	5.9	0	3.5	NR	NR	NR	NR
Boulet	2019	NR	NR	NR	NR	NR	NR	NR	NR	NR	NR	71
Chu	2011	29.6	0	51.3		8.1	0	11.1	NR	NR	NR	NR
Dorresteijn	2002	NR	44.1	NR	NR	NR	24.8	31.1	NR	NR	NR	NR
Haynes	2009	NR	NR	NR	NR	NR	NR	NR	NR	NR	NR	NR
Kwon	2021	20.0	28.4	13.8	19.0	7.9	10.9		NR	NR	NR	NR
Lee	2011	100.0	0	0	0	0	0	NR	NR	NR	NR	NR
Lee	2020	10.8	31.0	10.4	20.3	8.2	11.2	7.9	NR	NR	NR	NR
Sun	2022	0	0	100	0	0	0	0	NR	NR	NR	NR
Sun	2023	1.4	35.5	36.2	19.6	5.3	0	2.1	NR	NR	NR	NR
van Aken	2021	4.0	45.0	36.0	6.0	9.0	0	0	NR	NR	NR	NR
Yeh	2022	0	3.5	0	93.1	0	3.1	0	25.4	16.5	11.8	45.3
Yip	2024	47.7	12.5	5.4	0.2	3.8	6.6	23.8	14.5	15.8	22.6	47.1

Abbreviations: HNC, head and neck cancer; NPC, nasopharyngeal cancer; NR, not reported.

### Stroke Incidence

**Figure 2:**
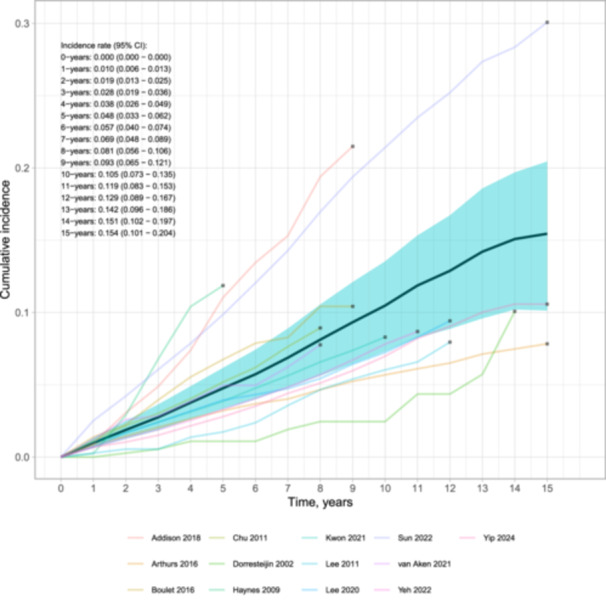
Individual patient time‐to‐event meta‐analysis of cumulative stroke risk among head and neck cancer patients. The solid and translucent turquoise lines represent the summary curve and 95% confidence intervals, respectively; black squares represent the last follow‐up time.

Stroke incidence was pooled from time‐to‐event IPD reconstructed from digitized survivor or failure curves. A total of 13 studies were included in the pooled IPD incidence. The findings revealed a notably high incidence of stroke among HNC patients at a rate of 1% per year, with 10% experiencing a stroke by 10 years and 15% by 15 years. The cumulative incidence curve is presented in Figure 2.

### Stroke Risk of HNC Compared to Healthy Controls

**Figure 3:**
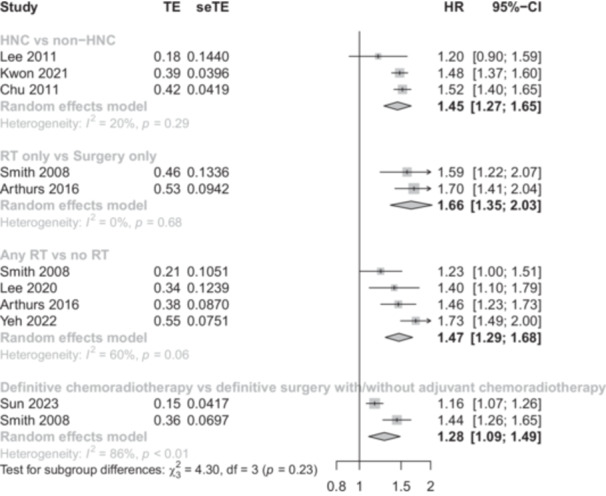
Pooled hazard ratio (HR) of risk of studies with different comparators. TE represents the estimate of treatment effect, and seTE represents the standard error of the treatment effect estimate.

Meta‐analyses of three studies were performed to evaluate the pooled HR of stroke in cohorts with HNC compared to healthy controls matched for age, gender, and comorbidities, including hypertension, diabetes mellitus, dyslipidemia, coronary heart disease, and atrial fibrillation (n = 163,325). The meta‐analyses showed that the risk of stroke was increased in patients with HNC compared to healthy controls (HR = 1.45; 95% CI: 1.27‐1.65; *I* ^2^: 20%). The studies included in the analysis of pooled HR can be found in Figure 3.

### Comparing Stroke Risk Across Treatment Groups

Pairwise analyses were conducted to evaluate stroke risk among different treatment modalities for HNC, including (i) radiotherapy alone versus surgery alone (n = 20,931); (ii) any radiotherapy versus no radiotherapy (n = 70,713); and (iii) definitive chemoradiotherapy versus definitive surgery with or without adjuvant chemoradiotherapy (n = 42,759) (Figure 3).

The results showed a significantly higher risk of stroke in HNC patients treated with radiotherapy alone compared to those who underwent surgery alone (HR = 1.66; 95% CI: 1.35‐2.03; *I*²: 0%).

The analysis also indicated an elevated stroke risk in HNC patients who received any form of radiotherapy compared to those who had not received radiotherapy (HR = 1.47; 95% CI: 1.29‐1.68; *I*²: 60%). Furthermore, the risk of stroke was higher in HNC patients who underwent definitive chemoradiotherapy compared to those who received definitive surgery with or without adjuvant chemoradiotherapy (HR = 1.28; 95% CI: 1.09‐1.49; *I*²: 86%).

## Discussion

In this study, we report an elevated stroke incidence of 1% per year among patients with HNC, nearly 50% higher than that of healthy controls. Notably, radiotherapy was associated with a significantly higher stroke risk compared to other treatment modalities.

Previous studies have demonstrated an increased risk of stroke in patients with cancer, with Zhang et al reporting a risk ratio of 1.66 across different cancers and a significantly increased risk in patients with head and neck, hematologic, lung, pancreas, and stomach cancer.^37^ Turner et al also report an increased risk of stroke in cancer across patients with pancreatic, lung and HNC, with the risk of hemorrhagic stroke in HNC patients being 1.54.^38^ Regarding the incidence of stroke in HNC patients, Lun et al, a meta‐analysis, reported a 1.4% incidence of stroke in the first year of diagnosis, but did not investigate the incidence of stroke beyond the first year of diagnosis.^39^ Yip et al also reported a cumulative incidence of 3% at 5 years and 7% at 10 years after diagnosis of HNC.^13^ Although our findings corroborate with previous studies, our study is presently the most comprehensive evidence‐based synthesis on the risk of stroke in HNC. To the best of our knowledge, our study is the first to demonstrate a pooled HR of stroke in HNC patients and that stroke in HNC patients occurs at a rate of 1% per year.

The proposed mechanisms for the increased risk of stroke in cancer patients compared to healthy controls include a state of hypercoagulability,^40^ which is part of the Virchow's triad.^41^ This hypercoagulable state is thought to arise from neutrophil extracellular traps (NETs), which have been associated with arterial microthrombosis in cancer.^42^ NETosis has been demonstrated to stimulate chronic pathological inflammation, leading to tissue and DNA damage.^43^ Inflammation has also been recognized as a hallmark of cancer, with elevated markers such as tumor necrosis factor‐α, C‐reactive protein, and erythrocyte sedimentation rate commonly observed in cancer patients, likely due to the secretion of inflammatory cytokines by cancer‐associated fibroblasts.^44^ This persistent inflammation can lead to endothelial dysfunction, increasing the risk of thrombosis and ischemic stroke.

Although previous research has focused on the increased risk of venous thromboembolism in cancer patients,^45^ there is a gap in the understanding of the mechanisms underpinning arterial thrombosis and ischemic stroke. To address this, future studies should investigate arterial thrombosis in HNC patients, with a focus on potential mechanisms including NETs and their associated markers.^42^

We also found that the risk of stroke was notably higher in HNC patients who underwent radiotherapy, likely due to vascular injury that can lead to accelerated atherosclerosis. Radiotherapy may cause endothelial injury to the tunica intima of arteries,^46^ which is considered the initiating step of atherosclerosis.^47^ This injury triggers the release of cytokines, leading to platelet and macrophage aggregation and smooth muscle cell recruitment. Macrophages absorb oxidized low‐density lipoprotein (LDL) cholesterol, forming foam cells, whereas smooth muscle cells synthesize a fibrous cap that encases the lipid core, creating an atheroma. Over time, the atheroma may rupture, activating clotting factors and forming clots that can embolize to cerebral arteries, thus increasing stroke risk.^47^

Although our meta‐analyses did not assess specific stroke subtypes, such as large artery atherosclerosis linked to carotid stenosis, existing literature indicates that carotid stenosis is notably prevalent in HNC patients following radiotherapy.^48^ Despite this, routine screening for carotid stenosis is not currently recommended.^49^ This raises the question of whether regular screening and surveillance for carotid artery stenosis could be beneficial for HNC patients post‐radiotherapy, given their elevated risk of stroke. Future prospective cohort studies are necessary to investigate this further.

Furthermore, we demonstrated that cohorts of HNC patients undergoing chemoradiotherapy had a significantly increased risk of stroke. Although studies have shown that chemoradiotherapy in HNC patients reported better outcomes compared to radiotherapy alone,^50^ the association of increased stroke risk in HNC patients receiving chemoradiotherapy should not be overlooked. The potential mechanism for increased stroke risk in HNC patients undergoing chemotherapy^51^ may be due to endothelial toxicity and abnormalities of coagulation factors,^52^ caused by antineoplastic agents such as cisplatin, l‐asparaginase, and so forth. This increases the risk of cerebral arterial or venous thrombosis.^53^ Chemotherapy can result in thrombocytopenia^54^ in cancer patients, hence increasing the risk of hemorrhagic stroke in patients. Chemotherapy can also by itself result in intracranial hemorrhage mediated by hemorrhagic vasculitis or cerebritis.^55^ Ischemic stroke occurs more often particularly after cisplatin‐based chemotherapy,^56^ via a multifaceted pathway involving vasospasm. Vasospasm arises due to electrolyte imbalance, endothelial dysfunction, platelet hyperaggregation, and other mechanisms.^56^ In contrast, several studies have reported that chemotherapy showed no association^57^ with increased risk of stroke in cancer patients, and that the presumable increase in stroke risk was associated with more advanced stages of cancer. Rogers and colleagues discussed that there is only a speculative attribution^53^ of thrombosis to chemotherapy due to the unavailability of prospective studies to investigate other thrombotic causes. Further research is needed to elucidate the relationship between chemotherapy and stroke in HNC patients.

## Limitations

Our study has several limitations. Despite the methodological rigor of the meta‐analysis, some heterogeneity was observed. We lacked IPD, which prevented us from accounting for variables such as the stage of HNC, stroke subtype, and patient lifestyle factors like diet, smoking, and exercise—key elements that could influence stroke risk. These factors may have acted as important confounders and potentially contributed to the observed increase in stroke risk.

Besides that, there was a small number of studies in individual subgroups, such as two studies in the subgroup comparing radiotherapy alone versus surgery alone; thus, these findings remain to be externally validated in a wider variety of populations.

Furthermore, we were unable to establish a dose‐dependent relationship between the treatment dose and risk of stroke in HNC patients due to heterogeneity and an insufficient number of studies. Out of the nine studies included in the meta‐analysis for the pooled risk of stroke in HNC patients, only two studies compared the risk of stroke in groups receiving different dosages of radiotherapy. Even so, the findings were vastly different, with Yeh et al suggesting that a dose of 60 to 80 Gray (Gy) resulted in lower risk of stroke compared to patients receiving a lower dosage of 0 to 60 Gy,^11^ whereas van Aken et al reported that 10 Gy of radiation to the carotid arteries is the main prognostic factor for increased risk of ischemic cerebrovascular events.^34^

Besides that, although we pooled the risk of stroke in HNC patients receiving radiotherapy as a primary treatment compared to those who had surgery only, we could not compare the risk of stroke in patients receiving radiotherapy as a primary treatment compared to those receiving radiotherapy as an adjunct. This adds to the heterogeneity, given that the dosage for patients receiving primary treatment is typically higher than in adjuvant treatment.

Given the limitations, future studies should thus investigate the risk of stroke in cohorts stratified by the dosage and type of radiotherapy, since certain more recently available modalities like proton therapy result in less collateral damage to surrounding tissue and may possibly result in a lower risk of stroke.^58^

We were also unable to pool the risk of stroke in patients with different types of HNC receiving radiotherapy, since the site of irradiation, whether to the primary site or the neck, and dose would differ as well. Nonetheless, we were still able to compare the stroke risk across the pre‐planned treatment groups. Future research should aim to include these crucial variables and explore dose‐dependent effects to enhance our understanding of stroke risk in this population.

Besides the heterogeneity in the IPD, there could also be heterogeneity in the curve reconstruction in the process of extracting the IPD. Any variations in reporting and curve resolution may potentially introduce bias and decrease the quality of the digitized survival curves. However, we tried to minimize bias by using the WebPlotDigitiser software and the algorithm developed by Guyot et al to extract the IPD. Subsequently, we also evaluated the accuracy of reconstruction using published methods.^16^

Furthermore, due to the nature of our statistical analysis, we were unable to account for the competing risk of mortality. As the competing event of death was not accounted for, the incidence of stroke reported at 1‐year intervals relates to the probability of recovery for patients who remain alive before the time. This may overestimate the stroke probability in surviving patients. Future studies could investigate further on this by incorporating competing risk models to provide a clearer understanding of the interplay between stroke and mortality.

## Conclusion

Patients with HNC face an elevated incidence and increased risk of stroke, with radiotherapy posing a particularly significant risk compared to other treatment modalities. These findings highlight the critical need for targeted surveillance and the development of tailored preventive strategies to reduce stroke risk in this vulnerable population.

## Author Contributions

**Eda Liew**, data acquisition, quality control of data and algorithm, manuscript preparation, manuscript review, manuscript editing; **Jing Xuan Tan**, data acquisition, quality control of data and algorithm, manuscript preparation, manuscript review, manuscript editing; **Chen Ee Low**, quality control of data and algorithm, data analysis and interpretation, statistical analysis, manuscript preparation, manuscript review, manuscript editing; **Doreen Shu Lin Goh**, study design, data acquisition, quality control of data and algorithm, manuscript preparation, manuscript review, manuscript editing; **Esther Yanxin Gao**, data analysis and inerpretation, statistical analysis, manuscript editing; **Yao Hao Teo**, manuscript editing; **Emilie C.M. de Groot**, manuscript editing; **Jasper Senff**, manuscript editing; **Ching‐Hui Sia**, manuscript editing; **Leonard Leong Litt Yeo**, manuscript editing; **Anna See**, manuscript editing; **Benjamin Kye Jyn Tan**, study concepts, study design, data analysis and interpretation, statistical analysis, manuscript preparation, manuscript editing, manuscript review; **Benjamin Yong‐Qiang Tan**, study concepts, study design, manuscript preparation, manuscript editing, manuscript review.

## Disclosures

### Competing interests

None.

### Funding source

Leonard Leong Litt Yeo has received research funding from the NMRC, MOH, and NHIC, advisor to Seemode, Cortiro, and Sunbird‐bio, and has equity in Ceroflo (CSAINV23jul‐0002, TA19nov‐008). Benjamin Yong‐Qiang Tan has received research funding from the National Medical Research Council, Singapore (RTF23jul‐0014).

## Supporting information

Supporting Information.
