# Supplementary material for: Stroke Risk in Head and Neck Cancer: A Meta‐analysis of Reconstructed Individual Patient Survival Data
Source: Otolaryngol Head Neck Surg. 2025 Apr 7;173(1):17–26. doi: 10.1002/ohn.1249 (PMC12207361; doi:10.1002/ohn.1249)
Supplement: Supplementary file 1 — Supporting Information. [file OHN-173-17-s001.docx]

### Risk of Stroke in Patients with Head and Neck Cancer: A Systematic Review and Meta-Analysis

**Supplementary Figure 1:** Funnel plot for assessing publication bias in papers studying hazard ratio of stroke in patients with head & neck cancer, pooled HR

#### Supplementary Figure 2: Contour-enhanced funnel plot for assessing the association of stroke with head & neck cancer, with missing studies imputed via the trim-and-fill method

**Supplementary Figure 3:** Quantitative assessment of publication bias in papers studying hazard ratio of stroke in patients with head & neck cancer, pooled HR

#### Supplementary Figure 4: Outlier assessment of papers studying hazard ratio of stroke in patients with head & neck cancer, pooled HR

**Supplementary Figure 5:** Leave-one-out analysis of papers studying hazard ratio of stroke in patients with head & neck cancer, pooled HR

**Supplementary Figure 6:** Original and reconstructed Kaplan-Meier curves

**Supplementary Table 1:** Preferred Reporting Items of Systematic Reviews and Meta-analyses Checklist

**Supplementary Table 2:** Search Strategies

**Supplementary Table 3:** The Newcastle-Ottawa Scale (NOS) quality assessment of cohort studies

**Supplementary Table 4**: Main characteristics of the included studies

#### Supplementary Figure 1: Funnel plot for assessing publication bias in papers studying hazard ratio of stroke in patients with head & neck cancer, pooled HR


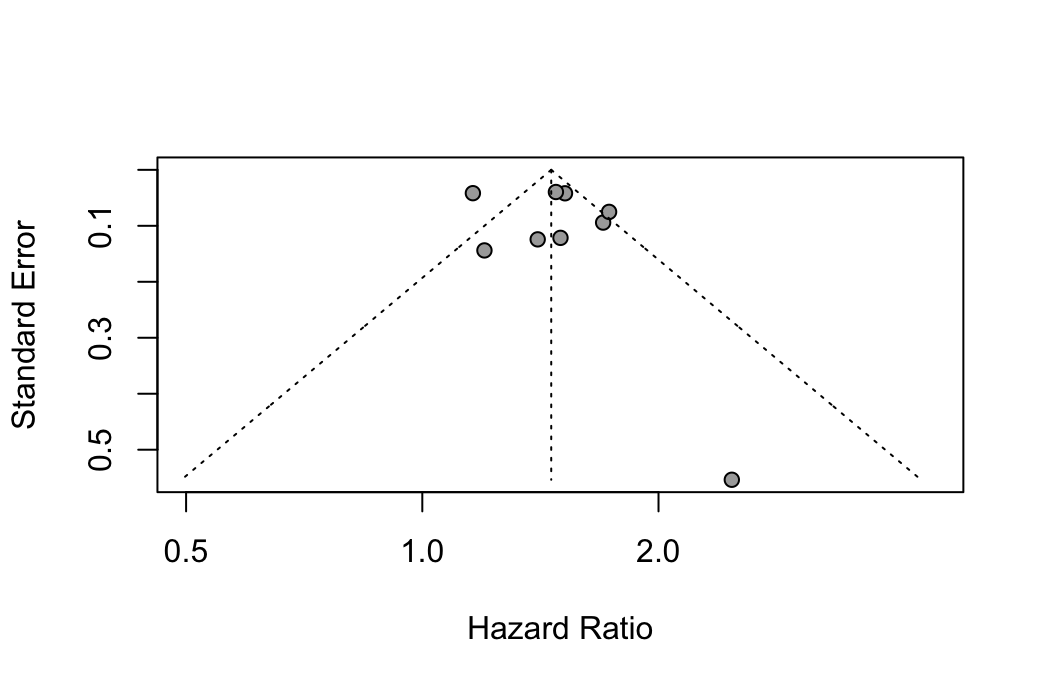


**Supplementary Figure 2**: Contour-enhanced funnel plot for assessing the association of stroke with head & neck cancer, with missing studies imputed via the trim-and-fill method

*Legend: Shaded circles represent the original study estimates and the unshaded circle represents the missing estimate imputed via the trim-and-fill method. Dark gray, gray and light gray contour lines indicate conventional milestones in levels of statistical significance (p<0.1, 0.05 and 0.01).*

#### Supplementary Figure 3: Quantitative assessment of publication bias in papers studying hazard ratio of stroke in patients with head & neck cancer, pooled HR


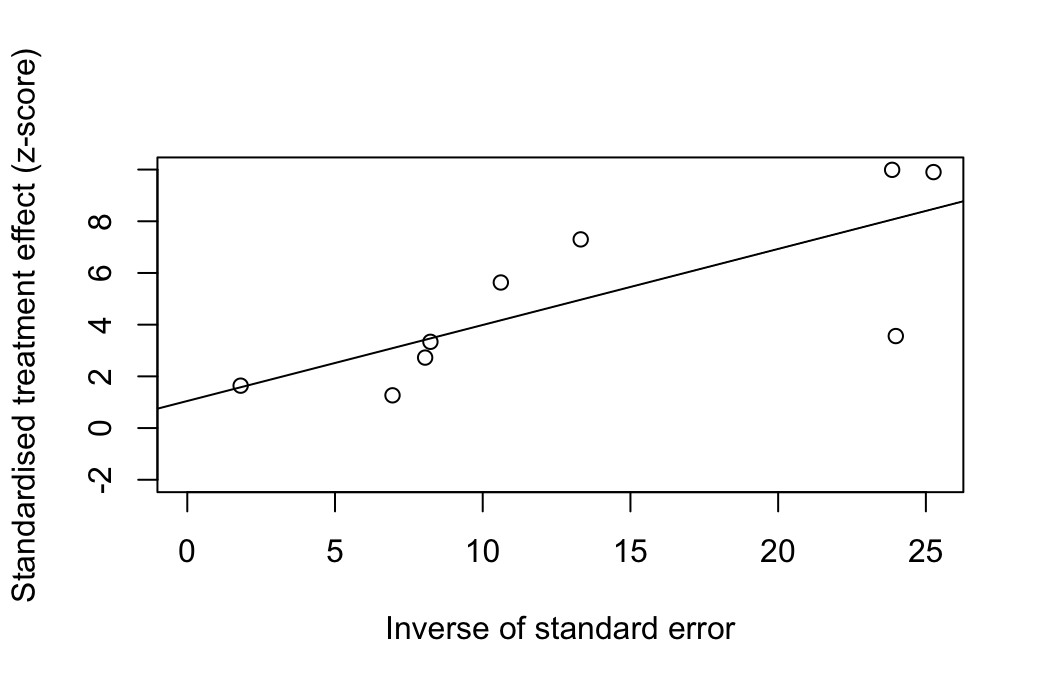


#### Supplementary Figure 4: Outlier assessment of papers studying hazard ratio of stroke in patients with head & neck cancer, pooled HR

*Legend: TE refers to the estimate of treatment effect, seTE refers to the standard error of treatment estimate (TE)*


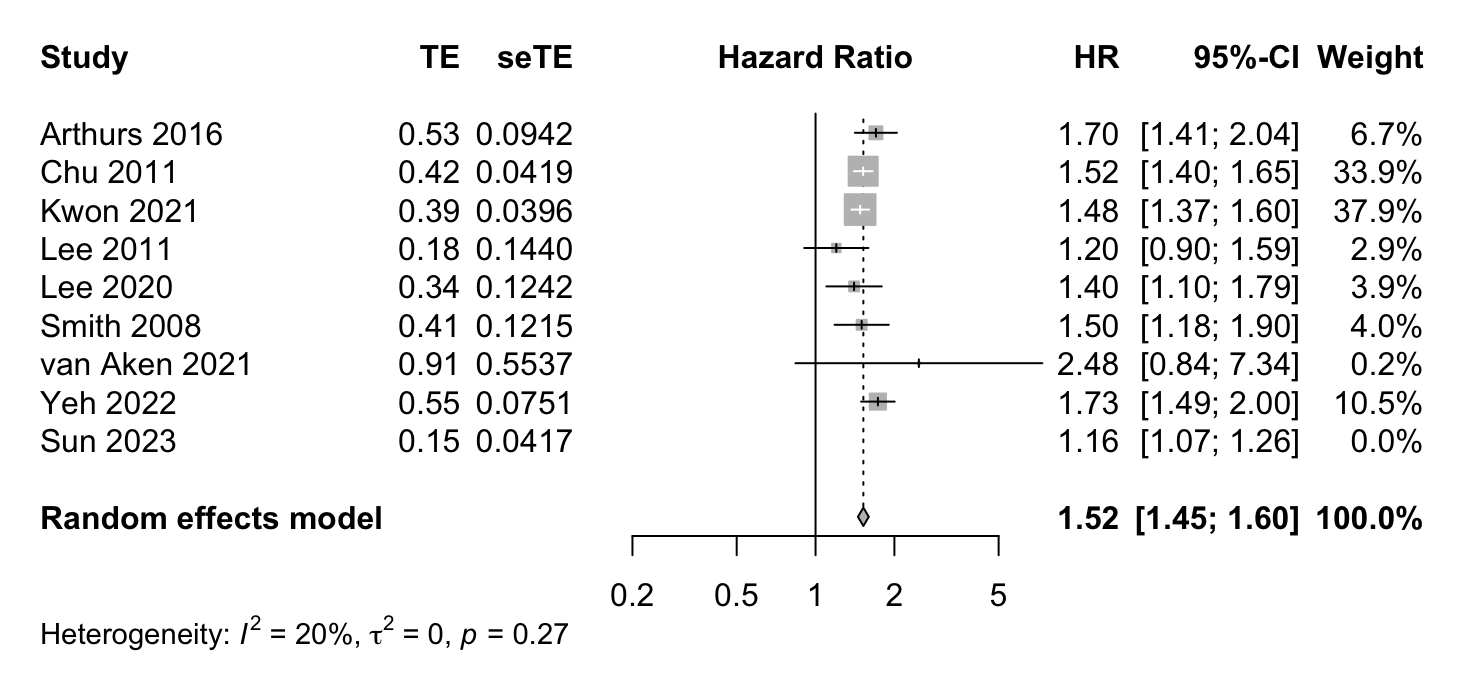


#### Supplementary Figure 5: Leave-one-out analysis of papers studying hazard ratio of stroke in patients with head & neck cancer, pooled HR


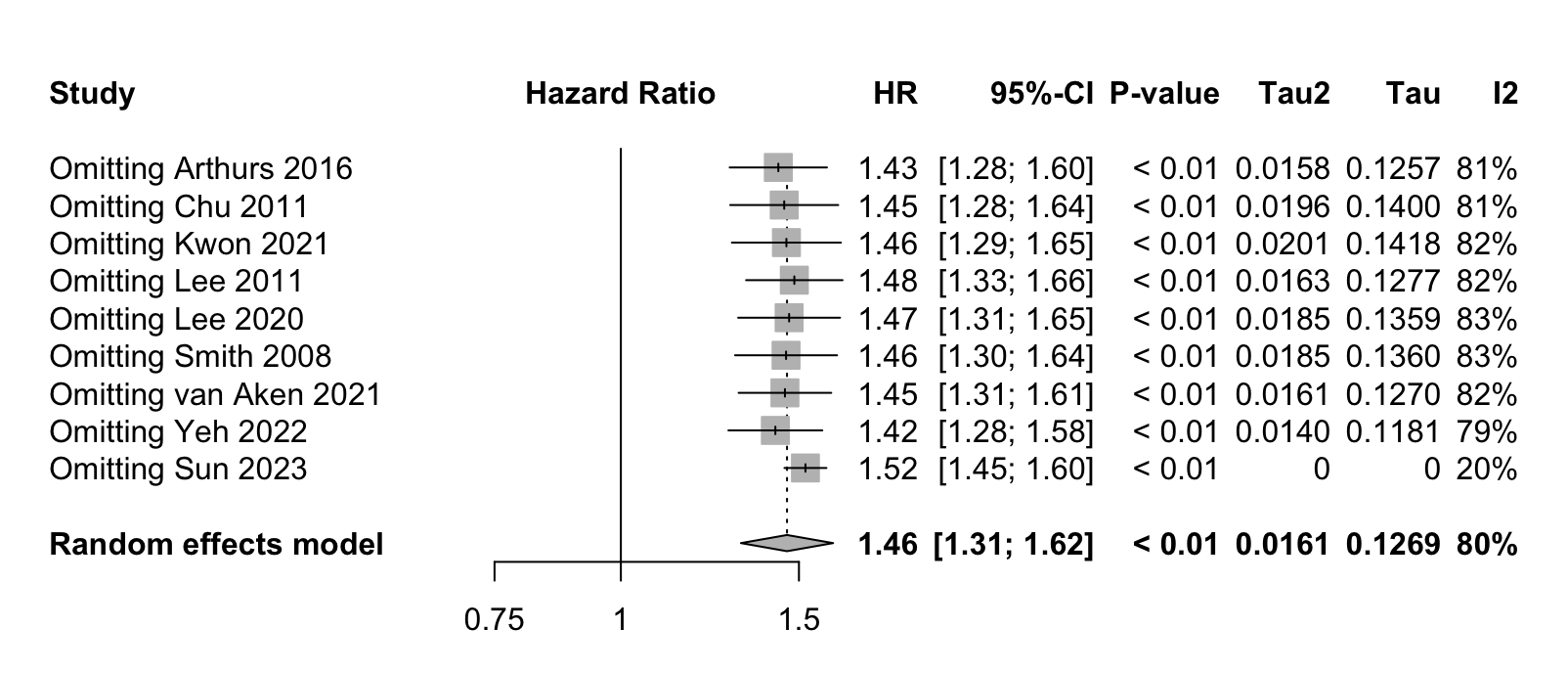


**Supplementary Figure 6:** Original and reconstructed Kaplan-Meier curves

| **Original curves** | **Reconstructed curves** |
| --- | --- |
| Addison 2018  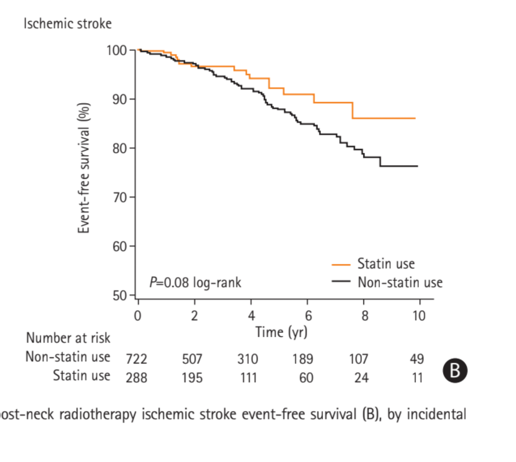 | \| Statin users \| Non-statin users \| \| --- \| --- \| \| 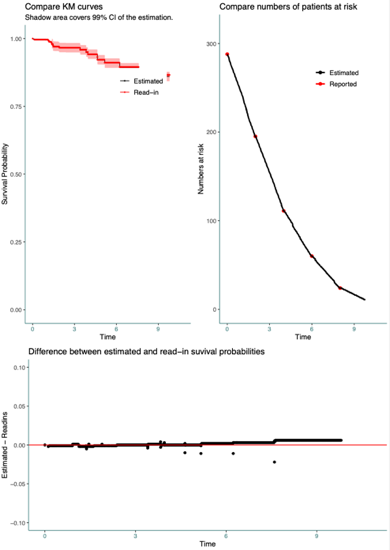 \| 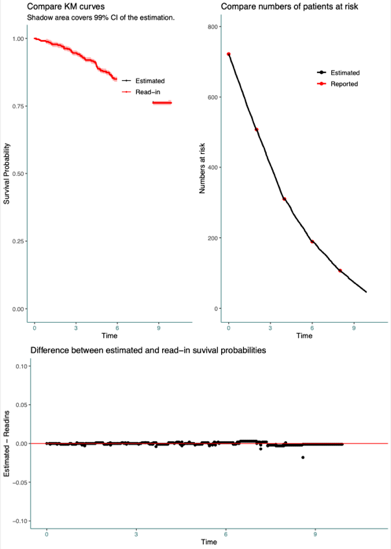 \| |
| Arthurs 2016  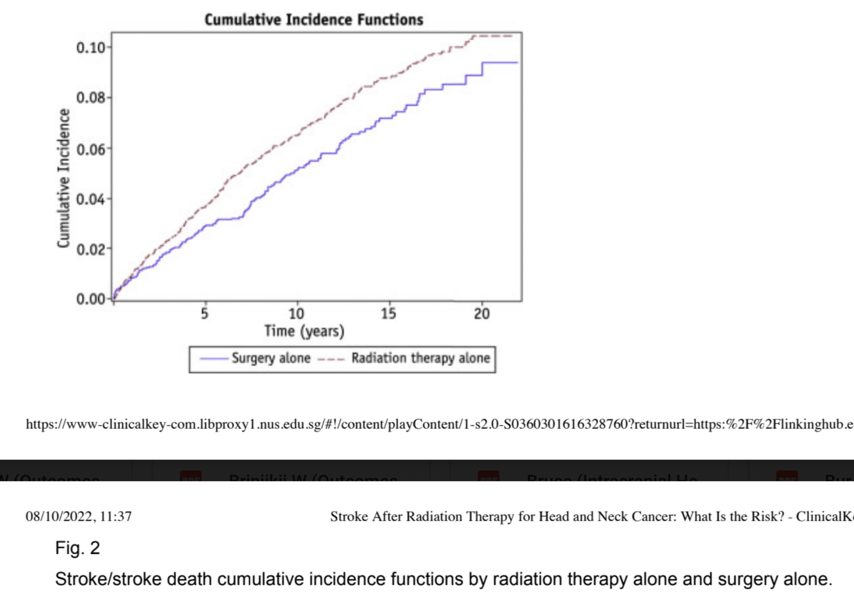 | \| RT alone \| Surgery alone \| \| --- \| --- \| \| 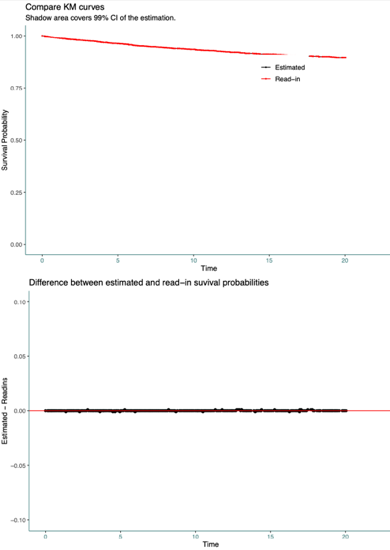 \| 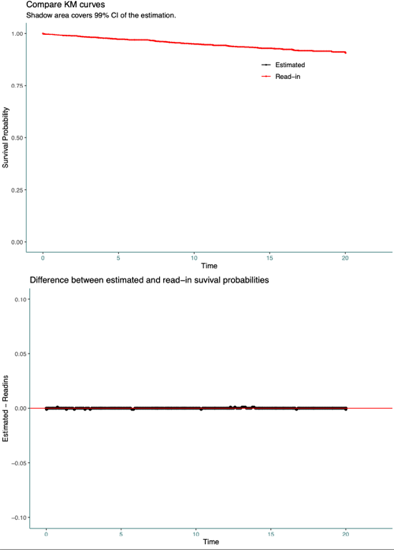 \| |
| Boulet 2016  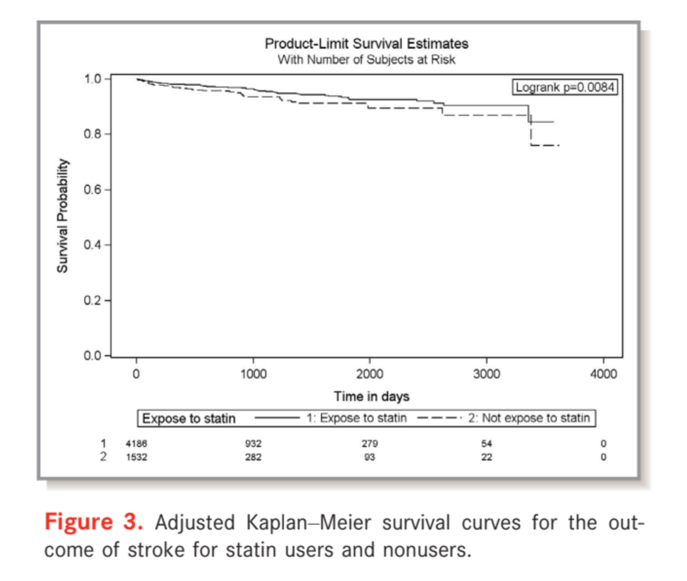 | \| Statin users \| Non-statin users \| \| --- \| --- \| \| 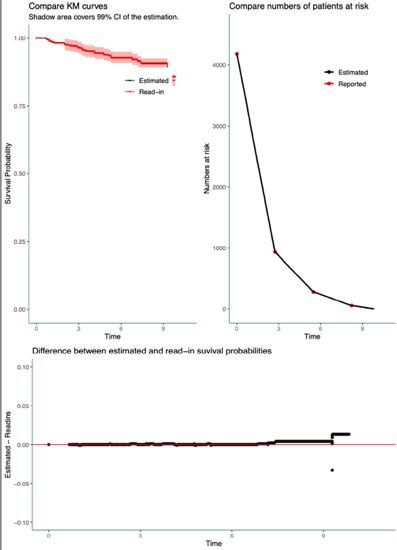 \| 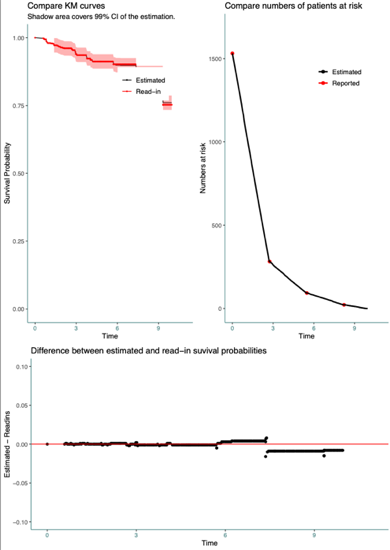 \| |
| Chu 2011  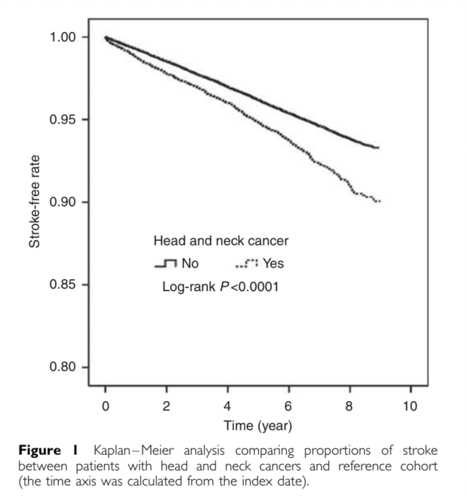 | \| General population \| HNC \| \| --- \| --- \| \| 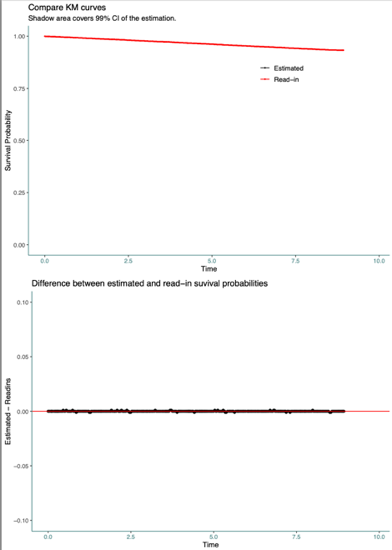 \| 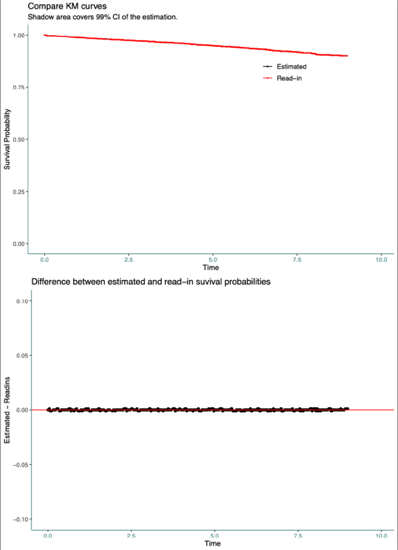 \| |
| Dorresteijin 2002  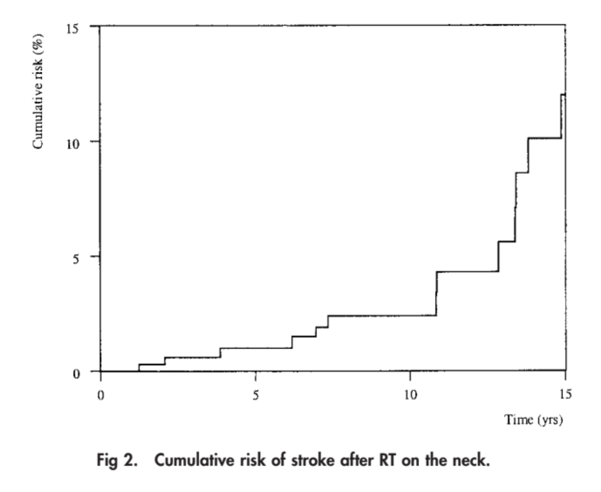 | 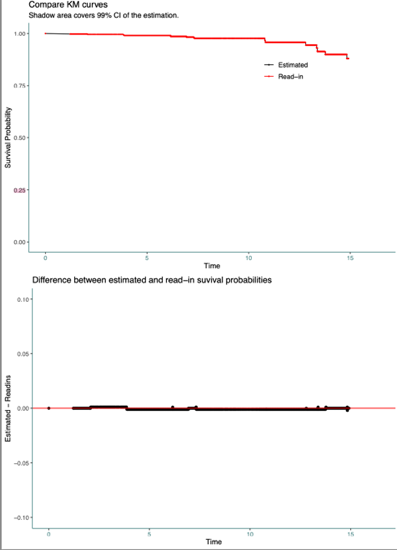 |
| Haynes 2009  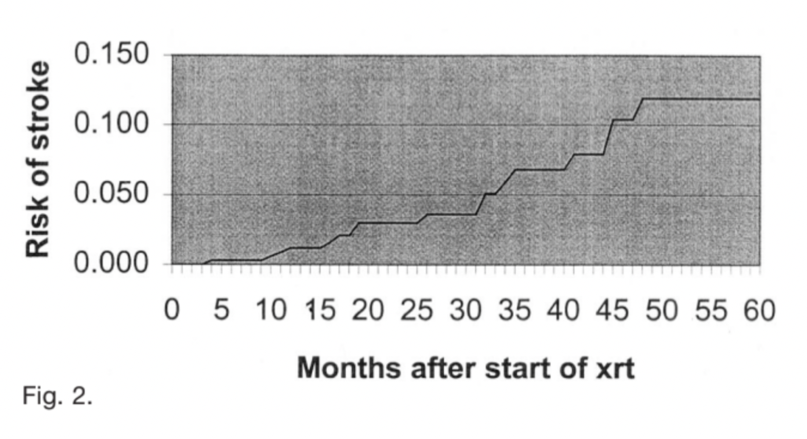 | 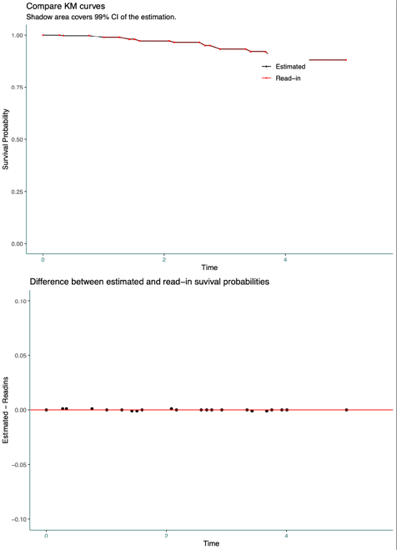 |
| Kwon 2021  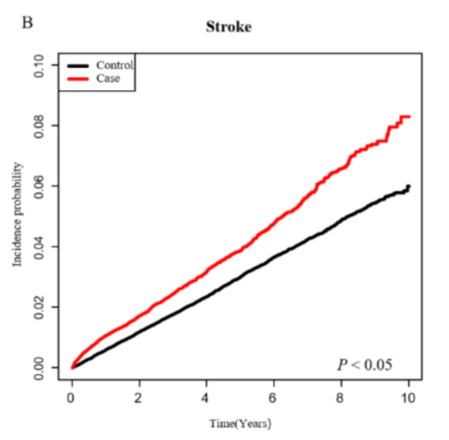 | \| General population \| HNC patients \| \| --- \| --- \| \| 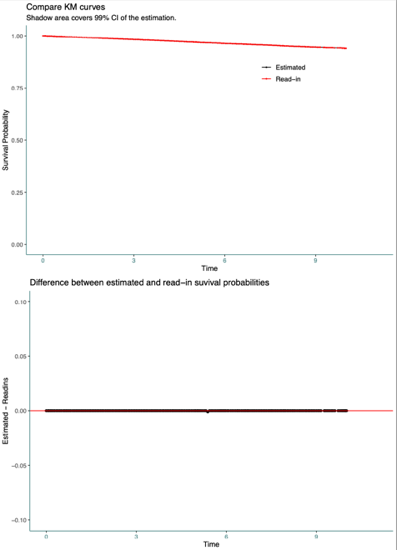 \| 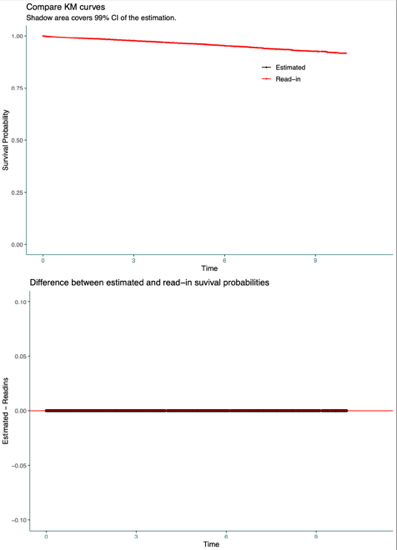 \| |
| Lee 2011   \| 35-54 years old \| 55-65 years old \| \| --- \| --- \| \| 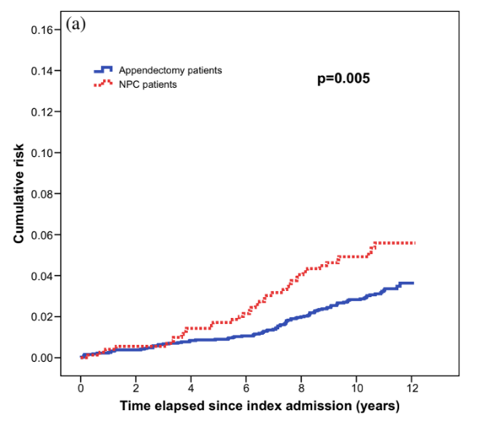 \| 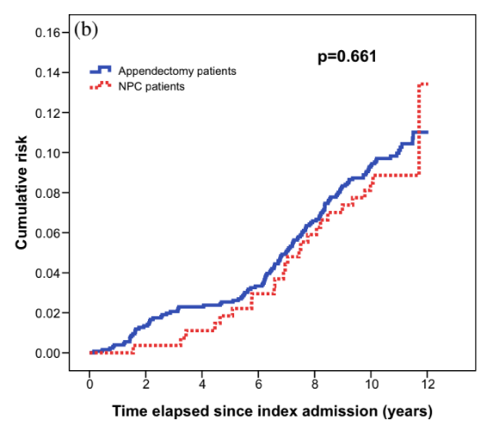 \| | \| 35-54 years old (appendectomy patients) \| 55-65 years old (appendectomy patients) \| \| --- \| --- \| \| 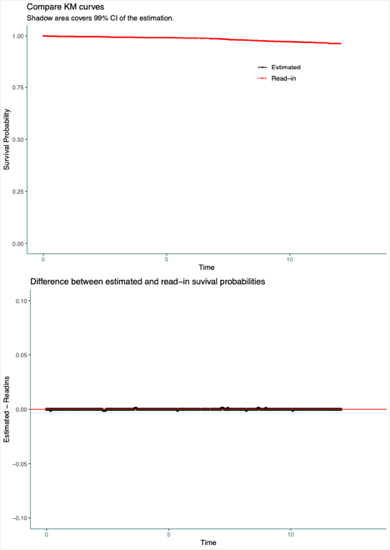 \| 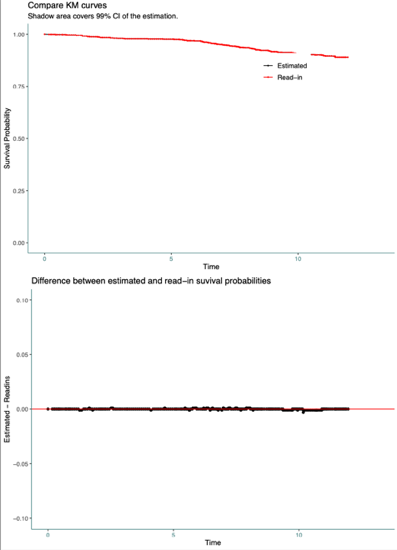 \| \| 35-54 years old (NPC patients) \| 55-65 years old (NPC patients) \| \| 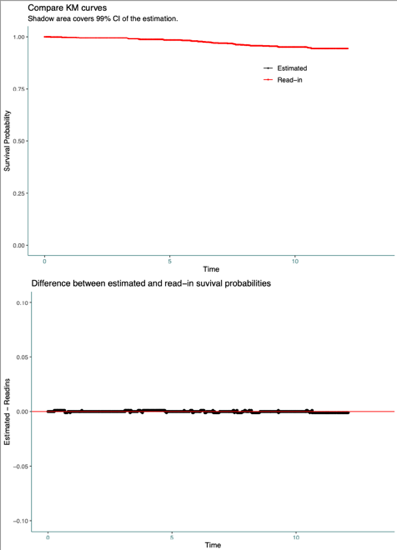 \| 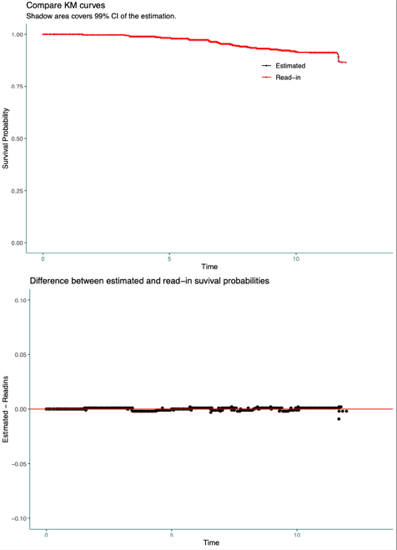 \| |
| Lee 2020  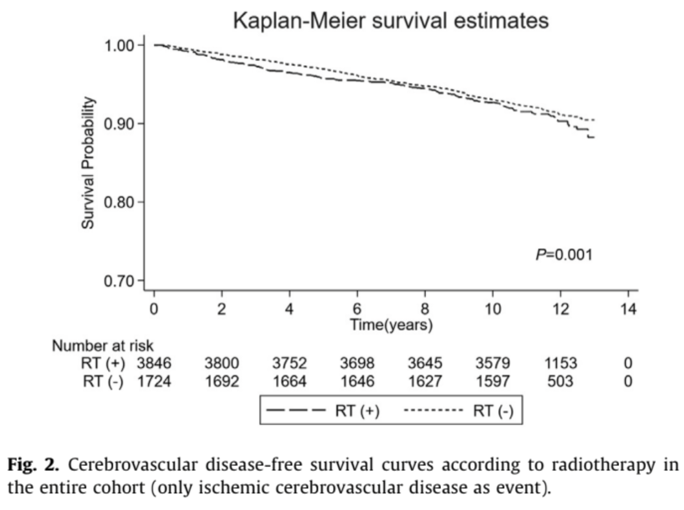 | \| Any RT \| No RT \| \| --- \| --- \| \| 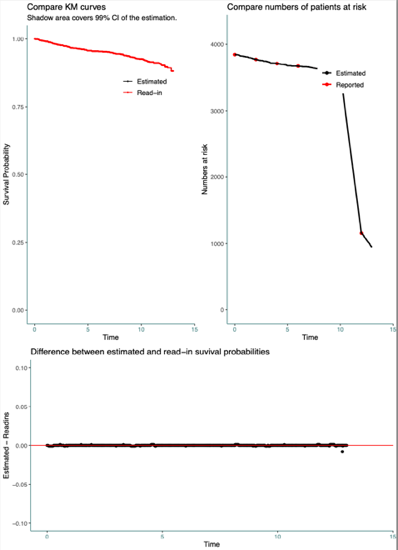 \| 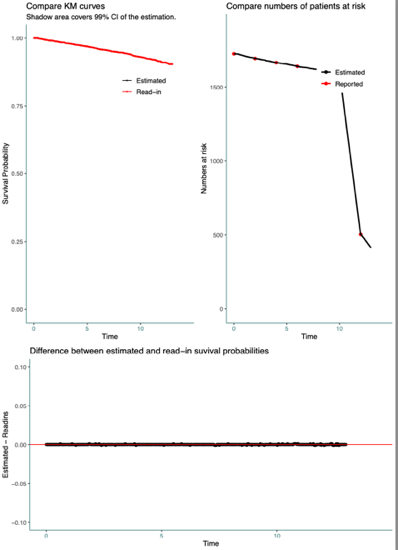 \| |
| Sun 2022  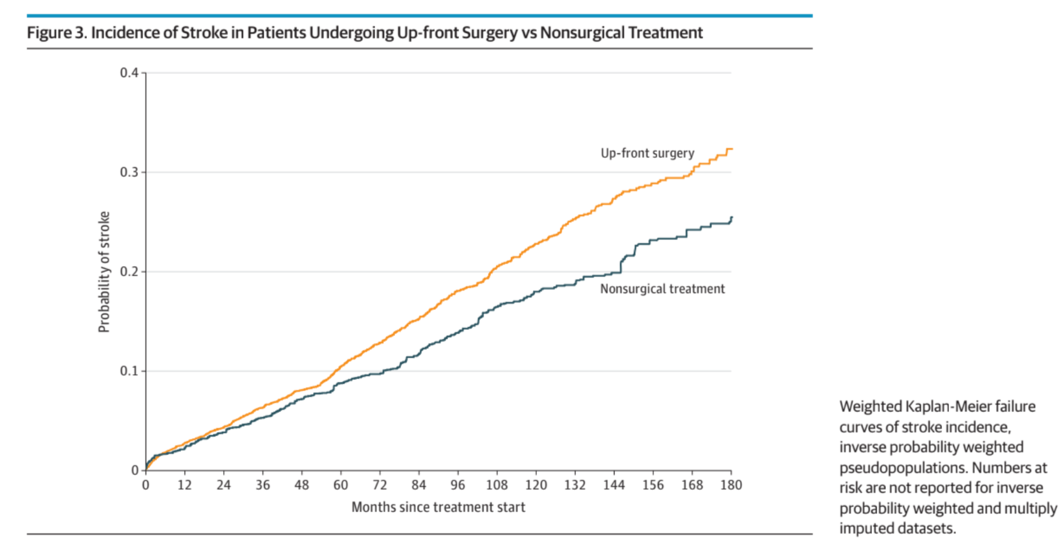 | \| Upfront surgery \| RT and/or CT \| \| --- \| --- \| \| 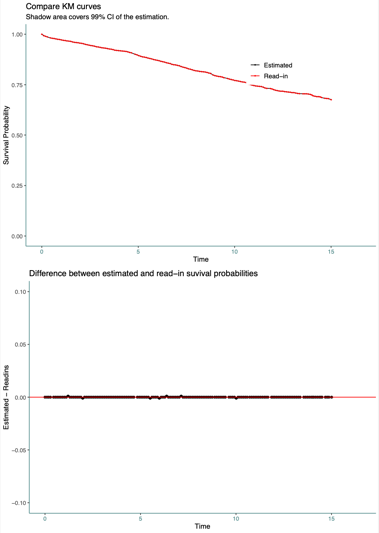 \| 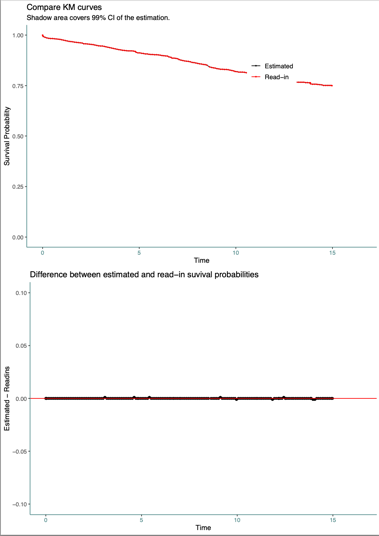 \| |
| van Aken 2021  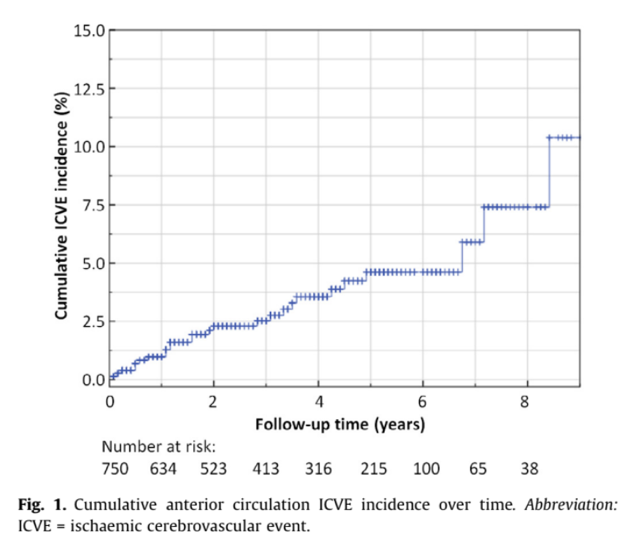 | 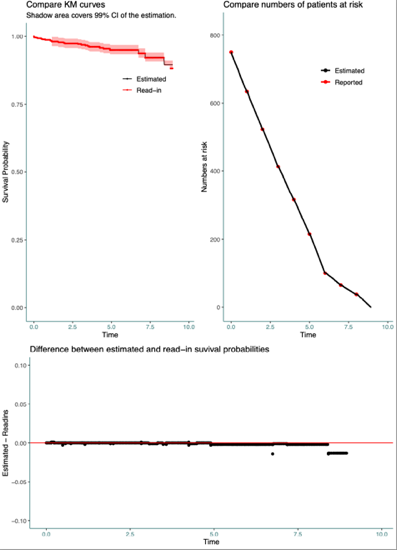 |
| Yeh 2022  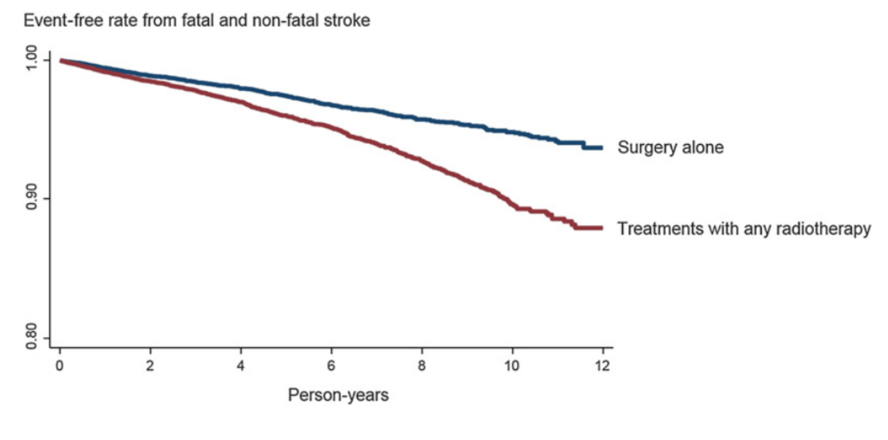 | \| Any RT \| Surgery alone \| \| --- \| --- \| \| 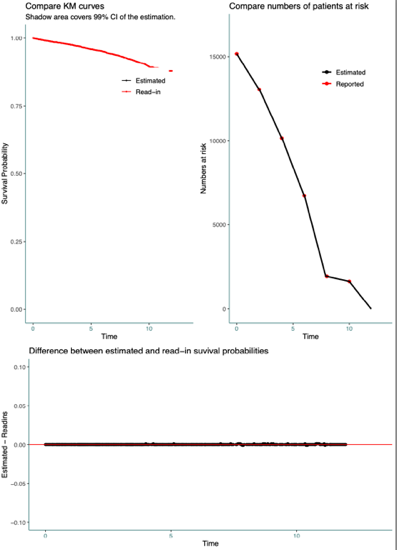 \| 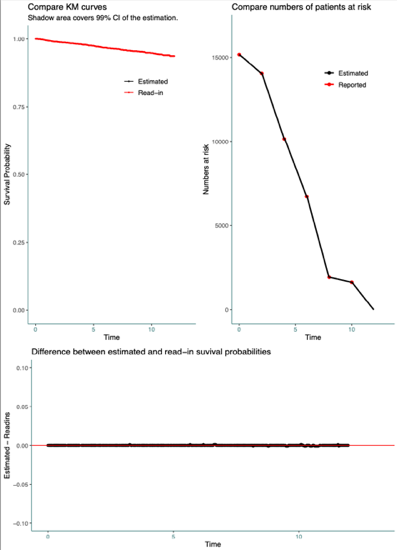 \| |
| Yip 2024  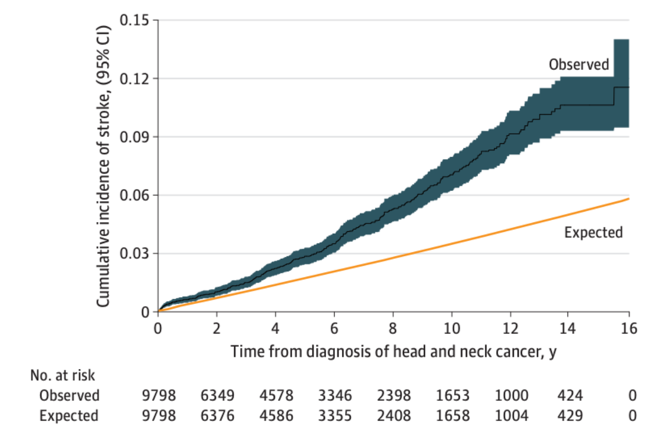 | 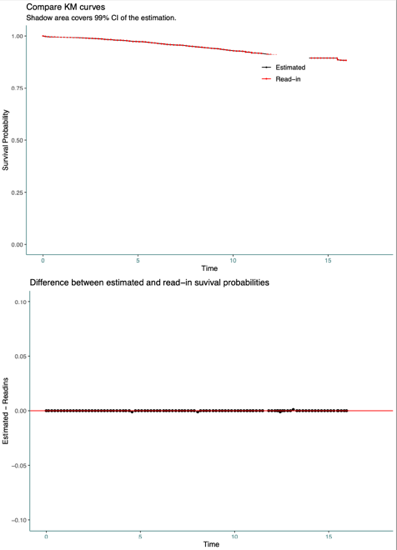 |

Abbreviations: HNC, head and neck cancer; RT, radiotherapy; CT, chemotherapy

**Supplementary Table 1:** Preferred Reporting Items of Systematic Reviews and Meta-analyses Checklist

| **Section and Topic** | **Item #** | **Checklist item** | **Location where item is reported** |
| --- | --- | --- | --- |
| **TITLE** | | |  |
| Title | 1 | Identify the report as a systematic review. | Page 1 of manuscript |
| **ABSTRACT** | | |  |
| Abstract | 2 | See the PRISMA 2020 for Abstracts checklist. | Page 4 of manuscript |
| **INTRODUCTION** | | |  |
| Rationale | 3 | Describe the rationale for the review in the context of existing knowledge. | Page 6 of manuscript |
| Objectives | 4 | Provide an explicit statement of the objective(s) or question(s) the review addresses. | Page 6 of manuscript |
| **METHODS** | | |  |
| Eligibility criteria | 5 | Specify the inclusion and exclusion criteria for the review and how studies were grouped for the syntheses. | Page 8 of manuscript |
| Information sources | 6 | Specify all databases, registers, websites, organisations, reference lists and other sources searched or consulted to identify studies. Specify the date when each source was last searched or consulted. | Page 8 of manuscript |
| Search strategy | 7 | Present the full search strategies for all databases, registers and websites, including any filters and limits used. | Supplementary Table 1 |
| Selection process | 8 | Specify the methods used to decide whether a study met the inclusion criteria of the review, including how many reviewers screened each record and each report retrieved, whether they worked independently, and if applicable, details of automation tools used in the process. | Page 8 of manuscript |
| Data collection process | 9 | Specify the methods used to collect data from reports, including how many reviewers collected data from each report, whether they worked independently, any processes for obtaining or confirming data from study investigators, and if applicable, details of automation tools used in the process. | Page 9 of manuscript |
| Data items | 10a | List and define all outcomes for which data were sought. Specify whether all results that were compatible with each outcome domain in each study were sought (e.g. for all measures, time points, analyses), and if not, the methods used to decide which results to collect. | Page 9 of manuscript |
|  | 10b | List and define all other variables for which data were sought (e.g. participant and intervention characteristics, funding sources). Describe any assumptions made about any missing or unclear information. | Page 9 of manuscript |
| Study risk of bias assessment | 11 | Specify the methods used to assess risk of bias in the included studies, including details of the tool(s) used, how many reviewers assessed each study and whether they worked independently, and if applicable, details of automation tools used in the process. | Page 11 of manuscript |
| Effect measures | 12 | Specify for each outcome the effect measure(s) (e.g. risk ratio, mean difference) used in the synthesis or presentation of results. | Page 9 of manuscript |
| Synthesis methods | 13a | Describe the processes used to decide which studies were eligible for each synthesis (e.g. tabulating the study intervention characteristics and comparing against the planned groups for each synthesis (item #5)). | Page 9 of manuscript |
|  | 13b | Describe any methods required to prepare the data for presentation or synthesis, such as handling of missing summary statistics, or data conversions. | Page 9 of manuscript |
|  | 13c | Describe any methods used to tabulate or visually display results of individual studies and syntheses. | Page 9 of manuscript |
|  | 13d | Describe any methods used to synthesize results and provide a rationale for the choice(s). If meta-analysis was performed, describe the model(s), method(s) to identify the presence and extent of statistical heterogeneity, and software package(s) used. | Page 9 of manuscript |
|  | 13e | Describe any methods used to explore possible causes of heterogeneity among study results (e.g. subgroup analysis, meta-regression). | Page 9 of manuscript |
|  | 13f | Describe any sensitivity analyses conducted to assess robustness of the synthesized results. | Page 9 of manuscript |
| Reporting bias assessment | 14 | Describe any methods used to assess risk of bias due to missing results in a synthesis (arising from reporting biases). | Page 12 of manuscript |
| Certainty assessment | 15 | Describe any methods used to assess certainty (or confidence) in the body of evidence for an outcome. | Page 12 of manuscript |
| **RESULTS** | | |  |
| Study selection | 16a | Describe the results of the search and selection process, from the number of records identified in the search to the number of studies included in the review, ideally using a flow diagram. | Figure 1 |
|  | 16b | Cite studies that might appear to meet the inclusion criteria, but which were excluded, and explain why they were excluded. | Page 12 of manuscript |
| Study characteristics | 17 | Cite each included study and present its characteristics. | Page 12 of manuscript |
| Risk of bias in studies | 18 | Present assessments of risk of bias for each included study. | Supplementary Table 2 |
| Results of individual studies | 19 | For all outcomes, present, for each study: (a) summary statistics for each group (where appropriate) and (b) an effect estimate and its precision (e.g. confidence/credible interval), ideally using structured tables or plots. | Table 1 |
| Results of syntheses | 20a | For each synthesis, briefly summarise the characteristics and risk of bias among contributing studies. | Page 12 of manuscript |
|  | 20b | Present results of all statistical syntheses conducted. If meta-analysis was done, present for each the summary estimate and its precision (e.g. confidence/credible interval) and measures of statistical heterogeneity. If comparing groups, describe the direction of the effect. | Page 12 of manuscript |
|  | 20c | Present results of all investigations of possible causes of heterogeneity among study results. | Page 12 of manuscript |
|  | 20d | Present results of all sensitivity analyses conducted to assess the robustness of the synthesized results. | Page 12 of manuscript |
| Reporting biases | 21 | Present assessments of risk of bias due to missing results (arising from reporting biases) for each synthesis assessed. | Page 12 of manuscript |
| Certainty of evidence | 22 | Present assessments of certainty (or confidence) in the body of evidence for each outcome assessed. | Page 12 of manuscript |
| **DISCUSSION** | | |  |
| Discussion | 23a | Provide a general interpretation of the results in the context of other evidence. | Page 15 of manuscript |
|  | 23b | Discuss any limitations of the evidence included in the review. | Page 19 of manuscript |
|  | 23c | Discuss any limitations of the review processes used. | Page 19 of manuscript |
|  | 23d | Discuss implications of the results for practice, policy, and future research. | Page 19 of manuscript |
| **OTHER INFORMATION** | | |  |
| Registration and protocol | 24a | Provide registration information for the review, including register name and registration number, or state that the review was not registered. | Page 8 of manuscript |
|  | 24b | Indicate where the review protocol can be accessed, or state that a protocol was not prepared. | Page 8 of manuscript |
|  | 24c | Describe and explain any amendments to information provided at registration or in the protocol. | NA |
| Support | 25 | Describe sources of financial or non-financial support for the review, and the role of the funders or sponsors in the review. | Page 2 of manuscript |
| Competing interests | 26 | Declare any competing interests of review authors. | Page 2 of manuscript |
| Availability of data, code and other materials | 27 | Report which of the following are publicly available and where they can be found: template data collection forms; data extracted from included studies; data used for all analyses; analytic code; any other materials used in the review. | Page 2 of manuscript |

**Supplementary Table 2:** Search Strategies

| ​​**Database** | **​Search Strategy** | **​Number of articles retrieved** |
| --- | --- | --- |
| ​PubMed | 1. ("head and neck"[Title/Abstract] OR "nasopharyngeal"[Title/Abstract] OR "laryngeal"[Title/Abstract] OR "oral"[Title/Abstract] OR "pharyngeal"[Title/Abstract] OR "oropharyngeal"[Title/Abstract] OR "tongue"[Title/Abstract] OR "buccal"[Title/Abstract] OR "mouth"[Title/Abstract] OR "tonsillar"[Title/Abstract]) AND ("cancer*"[Title/Abstract] OR "carcinoma*"[Title/Abstract] OR "neoplasm*"[Title/Abstract] OR "tumor*"[Title/Abstract] OR "tumour*"[Title/Abstract]) 2. "Head and Neck Neoplasms"[MeSH Terms] OR "Squamous Cell Carcinoma of Head and Neck"[MeSH Terms] 3. "Ischemic Stroke"[MeSH Terms] OR "Ischaemic stroke"[Title/Abstract] OR "Brain ischemia"[Title/Abstract] OR "brain infarct*"[Title/Abstract] OR "brain thrombo*"[Title/Abstract] OR "brain emboli*"[Title/Abstract] OR "brain occlus*"[Title/Abstract] OR "ischemic attack, transient"[MeSH Terms] OR "Transient ischemic attack"[Title/Abstract] OR "transient attack"[Title/Abstract] OR "TIA"[Title/Abstract] OR "cerebrovascular disease"[Title/Abstract] OR "cerebrovascular accident"[Title/Abstract] OR "stroke"[Title/Abstract] OR "strokes"[Title/Abstract] OR "large vessel occlusion"[Title/Abstract] OR "Brain ischemia"[Title/Abstract] OR "Brain ischaemia"[Title/Abstract] OR "brain infarction"[Title/Abstract] OR "cerebral infarction"[Title/Abstract] OR "cerebral ischemia"[Title/Abstract] OR "cerebral ischaemia"[Title/Abstract]   ​ | ​1784 |
| ​EMBASE | 1. ('head and neck':ti,ab OR nasopharyngeal:ti,ab OR laryngeal:ti,ab OR oral:ti,ab OR pharyngeal:ti,ab OR oropharyngeal:ti,ab OR tongue:ti,ab OR buccal:ti,ab OR mouth:ti,ab OR tonsillar:ti,ab) AND ('cancer'/exp OR 'carcinoma'/exp OR 'neoplasm'/exp OR 'tumor'/exp OR 'tumour'/exp) 2. 'ischemic stroke'/exp OR 'ischaemic stroke':ti,ab OR 'brain infarct*':ti,ab OR 'brain thrombo*':ti,ab OR 'brain emboli*':ti,ab OR 'brain occlus*':ti,ab OR 'ischemic attack, transient'/exp OR 'transient ischemic attack':ti,ab OR 'transient attack':ti,ab OR 'tia':ti,ab OR 'cerebrovascular disease':ti,ab OR 'cerebrovascular accident':ti,ab OR 'stroke':ti,ab OR 'strokes':ti,ab OR 'large vessel occlusion':ti,ab OR 'brain ischemia':ti,ab OR 'brain ischaemia':ti,ab OR 'brain infarction':ti,ab OR 'cerebral infarction':ti,ab OR 'cerebral ischemia':ti,ab OR 'cerebral ischaemia':ti,ab | ​1863 |
| ​SCOPUS | ​( TITLE-ABS-KEY ( "Ischemic Stroke"  OR  "Ischaemic stroke"  OR  "Brain ischemia"  OR  "brain infarct*"  OR  "brain thrombo*"  OR  "brain emboli*"  OR  "brain occlus*"  OR  "ischemic attack, transient"  OR  "Transient ischemic attack"  OR  "transient attack"  OR  "TIA"  OR  "cerebrovascular disease"  OR  "cerebrovascular accident"  OR  "stroke"  OR  "strokes"  OR  "large vessel occlusion"  OR  "Brain ischemia"  OR  "Brain ischaemia"  OR  "brain infarction"  OR  "cerebral infarction"  OR  "cerebral ischemia"  OR  "cerebral ischaemia" )  AND  TITLE-ABS-KEY ( ( "head and neck"  OR  "nasopharyngeal"  OR  "laryngeal"  OR  "oral"  OR  "pharyngeal"  OR  "oropharyngeal"  OR  "tongue"  OR  "buccal"  OR  "mouth"  OR  "tonsillar" )  AND  ( "cancer*"  OR  "carcinoma*"  OR  "neoplasm*"  OR  "tumor*"  OR  "tumour*" ) ) )  AND NOT  INDEX ( medline ) | ​1508​ |

**Supplementary Table 3:** The Newcastle-Ottawa Scale (NOS) quality assessment of cohort studies

| **Authors, Country, Year** | **Selection** | | | | **Comparability** | **Outcome** | | | **AHRQ* standards** |
| --- | --- | --- | --- | --- | --- | --- | --- | --- | --- |
|  | Representativeness of the exposed cohort | Selection of the non-exposed cohort | Ascertainment of exposure | Demonstration that outcome of interest was not present at start of study | Comparability of cohorts on the basis of the design or analysis | Assessment of outcome | Was follow-up long enough for outcomes to occur | Adequacy of follow-up of cohorts (≤20% loss) |  |
| Addison et al., USA, 2018 | ☆ | ☆ | ☆ |  | ☆ | ☆ | ☆ | ☆ | Good |
| Arthurs et al., Canada, 2016 | ☆ | ☆ | ☆ | ☆ | ☆ | ☆ |  | ☆ | Good |
| Boulet et al., Canada, 2019 | ☆ |  | ☆ |  | ☆ | ☆ | ☆ | ☆ | Fair |
| Chu et al., Taiwan, 2011 | ☆ | ☆ | ☆ |  | ☆ |  | ☆ | ☆ | Good |
| Dorresteijn et al., Netherlands, 2002 | ☆ | ☆ | ☆ |  | ☆ | ☆ | ☆ | ☆ | Good |
| Haynes et al., USA, 2002 | ☆ |  | ☆ |  |  | ☆ | ☆ |  | Poor |
| Kwon et al., South Korea, 2021 | ☆ | ☆ | ☆ | ☆ | ☆ | ☆ | ☆ |  | Good |
| Lee et al., Taiwan, 2011 | ☆ |  | ☆ |  | ☆ | ☆ | ☆ |  | Fair |
| Lee et al., South Korea, 2020 | ☆ | ☆ | ☆ | ☆ | ☆ | ☆ | ☆ |  | Good |
| Smith et al., USA, 2008 | ☆ | ☆ | ☆ |  | ☆ | ☆ |  | ☆ | Good |
| Sun et al., USA, 2022 |  | ☆ | ☆ | ☆ | ☆ | ☆ | ☆ |  | Good |
| Sun et al., USA, 2023 |  | ☆ | ☆ | ☆ | ☆ | ☆ | ☆ |  | Good |
| van Aken et al., Netherlands, 2021 |  |  | ☆ | ☆ | ☆ | ☆ | ☆ |  | Fair |
| Yeh et al., Taiwan, 2022 | ☆ |  | ☆ |  | ☆ | ☆ | ☆ | ☆ | Fair |
| Yip et al., Singapore, 2024 | ☆ | ☆ | ☆ |  | ☆ | ☆ | ☆ | ☆ | Good |

*Agency for Health Research and Quality

Note: A study can be given a maximum of one star for each numbered item within the Selection and Outcome categories. A maximum of two stars can be given for Comparability.

Thresholds for converting the Newcastle-Ottawa scales to AHRQ standards (good, fair, and poor):

**Good quality:** 3 or 4 stars in selection domain AND 1 or 2 stars in comparability domain AND 2 or 3 stars in outcome/exposure domain

**Fair quality:** 2 stars in selection domain AND 1 or 2 stars in comparability domain AND 2 or 3 stars in outcome/exposure domain

**Poor quality:** 0 or 1 star in selection domain OR 0 stars in comparability domain OR 0 or 1 stars in outcome/exposure domain

**Summary of AHRQ results for included Cohort Studies**

| AHRQ Standards | Total Number of Cohort Studies |
| --- | --- |
| **Good quality** | 10 |
| **Fair quality** | 4 |
| **Poor quality** | 1 |
| **Total** | 15 |

**Supplementary Table 4**: Main characteristics of the included studies

| **Author** | **Publication year** | **Region of study** | **Cancer type** | **Gender (%)** | **Total number of participants** | **Mean age*, SD** | **Comorbidities (%)** | **Covariates adjusted for** | **Treatment modalities (%)** | **Control group characteristics** | **Duration of follow up** | **Time to stroke event** | **Study methodology and characteristics** |
| --- | --- | --- | --- | --- | --- | --- | --- | --- | --- | --- | --- | --- | --- |
| Addison | 2018 | USA | Various | Male (69.9%), Female (30.1%) | 1,011 | 59.4 (13.4) | HTN: 44.2%  DM: 11.9%  HLD: 27.5%  CVA: 6.9%  Smoking: 65.8%  AF: 4.2%  CKD: 2.5% | Age, gender, comorbidities, radiotherapy dose | Chemotherapy: 80.1%  Radiotherapy: 100%  Surgery: 55.5% | HNC patients who did not use statins | Median of 3.4 years | Statin: Median of 3.0 years  Non-statin: Median of 3.3 years | Retrospective population-based cohort study  involving participants recruited from Massachusetts General Hospital database (2002-2012) |
| Arthurs | 2016 | Canada | Various | Male (75.3%),  Female (24.7%) | 14,069 | NR | HTN: 21.4%  DM: 7.1%  IHD: 4.5%  AF: 0.6% | Age, gender, comorbidities, cancer site | Chemotherapy: 17.2% Radiotherapy: 77.8% Surgery: 56.9% | HNC patients who underwent surgery only | 10-20 years | NR | Retrospective population-based cohort study involving participants recruited from the Ontario Cancer Registry and regional cancer treatment centers (1990-2010) |
| Boulet | 2019 | Canada | Various | Male (54.6%),  Female (45.4%) | 5,718 | 75 (6.1) | HTN: 71.0%  DM: 30.0%  HLD: 62.0%  CVA: 10.0%  AF: 21.0%  CKD: 15.0% HF: 22.0% | Age, gender, comorbidities | Radiotherapy: 100% | HNC patients who did not use statins | NR | Statin: Mean of 1.5 years  Non-statin: Mean of 1.6 years | Retrospective population-based cohort study involving participants recruited from Quebec computerized health insurance databases (2000-2011) |
| Chu | 2011 | Taiwan | Various | Male (85%), Female (15%) | 66,907 | 50.1 (19.6) | HTN: 21.5%  DM: 18.5% | Age, gender, comorbidities | NR | Reference cohort matched for age, gender, comorbidity (HTN, DM or both) and free from any cancer or prior stroke | 5-7 years | NR | Retrospective population-based cohort study involving participants recruited from the Taiwan National Health Insurance (NHI) database (2000-2002) |
| Dorresteijn** | 2002 | Netherlands | Laryngeal: 44.1%  Salivary Gland: 24.8%  Others: 31.1% | Male (61%), Female (39%) | 367 | NR | HTN: 15.5%  DM: 3.8%  Smoking: 43.6% | Age, gender | Radiotherapy: 100% | General population matched for sex and age | Median of 7.7 years | Median of 10.9 years (1.3-21.0 years) | Retrospective population-based cohort study involving participants recruited from the  Netherlands Cancer Institute database (1997-1998) |
| Haynes | 2009 | USA | Various | NR | 413 | NR | HTN: 29.3%  DM: 10.5% | Age, gender, smoking status | Radiotherapy: 100% | General population from Stockholm detailed in another study in 1981 | 2-146 months | NR | Retrospective population-based cohort study involving participants recruited from the University of Pennsylvania Medical Centre database (1987-2000) |
| Kwon | 2021 | Korea | Various | Male (75.5%),  Female (24.5%) | 90,948 | 58.65 (13.22) | HTN: 33.14%  DM: 14.77%  HLD: 13.17% | Age, gender, comorbidities | Chemotherapy: 33.9% Radiotherapy: 48.1% Surgery: 27.5% | Reference cohort matched for age and gender, without HNC, prior stroke or MI | 5-10 years | Within 2 years | Retrospective population-based cohort study involving participants recruited from the Korean National Health Insurance Service (NHIS) (2007-2013) |
| Lee | 2011 | Taiwan | Nasopharyngeal: 100% | Male (73.9%), Female (26.1%) | 5,470 | NR | HTN: 3.2%  DM: 4.0%  HLD: 3.0%  IHD: 1.0%  AF: 0.1% | Age, gender, comorbidities, geographic region, urbanization level of residence,  socioeconomic status | Chemotherapy: 34.4% Radiotherapy: 100% Surgery: 8.5% | Patients hospitalised for appendectomy from 1997-1998 | 12 years | Median 6.8 years | Retrospective population-based cohort study involving participants recruited from Taiwan’s National Health Research Institute Database (NHIRD) (1997-2008) |
| Lee | 2020 | Korea | Various | Male (77.8%), Female (22.2%) | 5,570 | 57.5 (13.5) | HTN: 60.7%  DM: 54.8%  HLD: 47.3%  Smoking: 39.8% | Age, gender | Chemotherapy: 22% Radiotherapy: 23% Surgery: 86% | Propensity Score Matching (PSM) cohort with HNC patients who did not undergo radiotherapy | Median 7.3 years | RT: Mean of 6.0 years  Non-RT: Mean of 5.2 years | Retrospective population-based cohort study involving participants recruited from the Korean Central Cancer Registry data and Korean National Health Insurance Service (2003-2005) |
| Smith | 2008 | USA | Various | Male (54%) Female (46%) | 6,862 | 76 (7) | NR | Age, gender, race, comorbidities, chemotherapy, tumour sites with higher  likelihood of receiving radiotherapy to the bilateral neck, number of positive nodes | Chemotherapy: 18% Radiotherapy: 70% Surgery: 71% | HNC patients who underwent surgery | Median 2.4 years (1.4-4.4) | 5-10 years | Retrospective population-based cohort study involving participants recruited from the Surveillance, Epidemiology, and End Results (SEER) - Medicare cohort (1992-2002) |
| Sun*** | 2022 | USA | Oropharyngeal (100%) | Male (99%), Female (1%) | 10,436 | 61.3 (8.15) | HTN: 57.9%  DM: 20.6%  HLD: 48.8%  IHD: 19.9%  Smoking: 92.6%  AF: 1.6%  CKD: 4.0% | Age, gender, comorbidities, diagnosis year, T and N stage,  smoking sta-  tus, marital status, deprivation index | Chemotherapy: 73.6%  Radiotherapy: 92.5%  Surgery: 26.0% | HNC patients who underwent nonsurgical definitive therapy (chemoradiotherapy or radiotherapy) | NR | NR | Retrospective population-based cohort study involving participants recruited from the US Veterans Health Administration (2000-2020) |
| Sun*** | 2023 | USA | Various | Male (98.9%), Female (1.1%) | 35,857 | 63.3 (8.15) | HTN: 67.0%  DM: 22.3%  HLD: 51.4%  IHD: 25.6%  CVA: 18.1%  Smoking: 83.2%  AF: 5.7%  CKD: 2.1% | Age, gender, race, comorbidities, year of diagnosis, smoking status, pri-  mary cancer site, ECOG performance status, and T and N stage, receipt of radiotherapy / chemotherapy | Chemotherapy: 17.5%  Radiotherapy: 69% Surgery: 67.3% | HNC patients who did not receive radiotherapy and had no prior MI or stroke | NR | 5-10 years | Retrospective population-based cohort study involving participants recruited from the US Veterans Health Administration (2000-2020) |
| van Aken | 2021 | Netherlands | Various | Male (75%), Female (25%) | 750 | 63 | HTN: 31.0%  DM: 12.0%  CVA: 10.0%  Smoking: 88.0% | Age, gender, comorbidities | Chemotherapy: 32.3% Radiotherapy: 100% | HNC patients who did not undergo radiotherapy | Median 3.4 years | Median 1.9 years | Retrospective population-based cohort study involving participants recruited from University Medical Centre Groningen, Netherlands (2007-2016) |
| Yeh | 2022 | Taiwan | Various | Male (90.6%), Female (9.4%) | 2,041,266 | 54.1 (11.1) | HTN: 41.6%  DM: 18.5%  HLD: 20.8%  Smoking: 40.4%  AF: 1.3% | Age, gender, geographic region of  residence, and tumour characteristics (including tumour stage, grades,  and histology) | Radiotherapy: 65.7% Surgery: 34.3% | Individuals aged 20-85 years; unmatched | Median 3.9 years | Median 3.8 years | Retrospective population-based cohort study involving participants recruited from the Taiwan Cancer Registry (2007-2016) |
| Yip | 2024 | Singapore | Various | Male (73.1%), Female (26.9%) | 9,803 | 58.33 (14.09) | NR | Age, gender, race, ethnicity | Chemotherapy: 42.8%  Radiotherapy: 68.6%  Surgery:  28.2% | General population | Median of 42.5 months (15.0- 94.5) | Median of 55 months (23-94) | Cross sectional study involving participants recruited from the Singapore Cancer Registry, the Singapore Stroke Registry, and the Registry of Birth and Deaths (2005-2020) |

Abbreviations: HNC, Head and Neck Cancer; HTN, Hypertension; DM, Diabetes Mellitus; IHD, Ischaemic Heart Disease; AF, Atrial Fibrillation; HLD, Hyperlipidaemia; MI, Myocardial Infarction; CVA, Cerebrovascular Accident; CKD, Chronic Kidney Disease; NR, Not Reported; RT: Radiotherapy

*Mean (standard deviation, SD) reported unless otherwise specified

**Dorresteijn 2002 included 4 patients with pleomorphic adenoma treated with radiotherapy. The study was included due to the potential malignant transformation of pleomorphic adenomas and repeated local recurrences.

***Sun 2022 was included in the cumulative incidence analysis, while Sun 2023 was included in the meta-analysis.
